# Supplementary material for: Lack of PCSK6 Increases Flow-Mediated Outward Arterial Remodeling in Mice
Source: Cells. 2020 Apr 18;9(4):1009. doi: 10.3390/cells9041009 (PMC7225991; doi:10.3390/cells9041009)
Supplement: Supplementary file 1 [file cells-09-01009-s001.pdf]

Supplementary

Table S1. Baseline bodyweight and arterial geometry from WT and KO mice included in the myography experiment.

	WT	KO	*p*-value
Bodyweight (g)	30±0.94	28±0.60	0.062
Ultrasound biomicroscopy			
Systolic circumference (mm)	1.9±0.06	1.7±0.05	0.095
Diastolic circumference (mm)	1.7±0.05	1.4±0.05	**0.009**

Baseline characteristics at baseline for WT (n=10) and KO (n=10) mice. Statistical analysis was performed using Student T-test for parametric and Mann-Whitney test for non-parametric data. WT= C57Bl/6J. KO= Pcsk6$^{-/-}$.

Table S2. Wire myography parameters at optimal stretch in control animals in WT and KO mice.

	WT	KO	*p*-value
Right CCA			
Active tension (mN/mm)	0.45±0.04	0.54±0.07	0.31
Circumference (mm)	1.52±0.05	1.42±0.03	0.11
Thoracic aorta			
Active tension (mN/mm)	1.36±0.21	1.53±0.09	0.70
Circumference (mm)	3.64±0.25	3.51±0.18	0.75

Comparison of control groups between WT (n=4) and KO (n=4) mice. Statistical analysis was performed using Mann-Whitney test for non-parametric data. WT= C57Bl/6J. KO= Pcsk6$^{-/-}$.

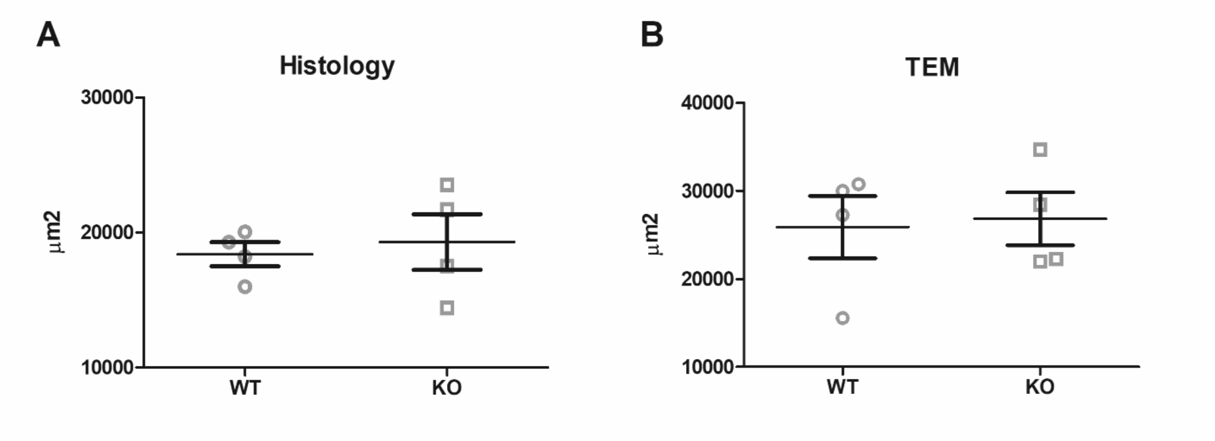

Figure S1. Difference in media area in right common carotid arteries exposed to increased flow. Morphometric analysis was performed on histochemical stainings (WT n=4, KO n=4) and TEM (WT n=4, KO n=4). KO= Pcsk6$^{-/-}$ mice. TEM= transmission electron microscopy. WT= C57Bl/6J mice.

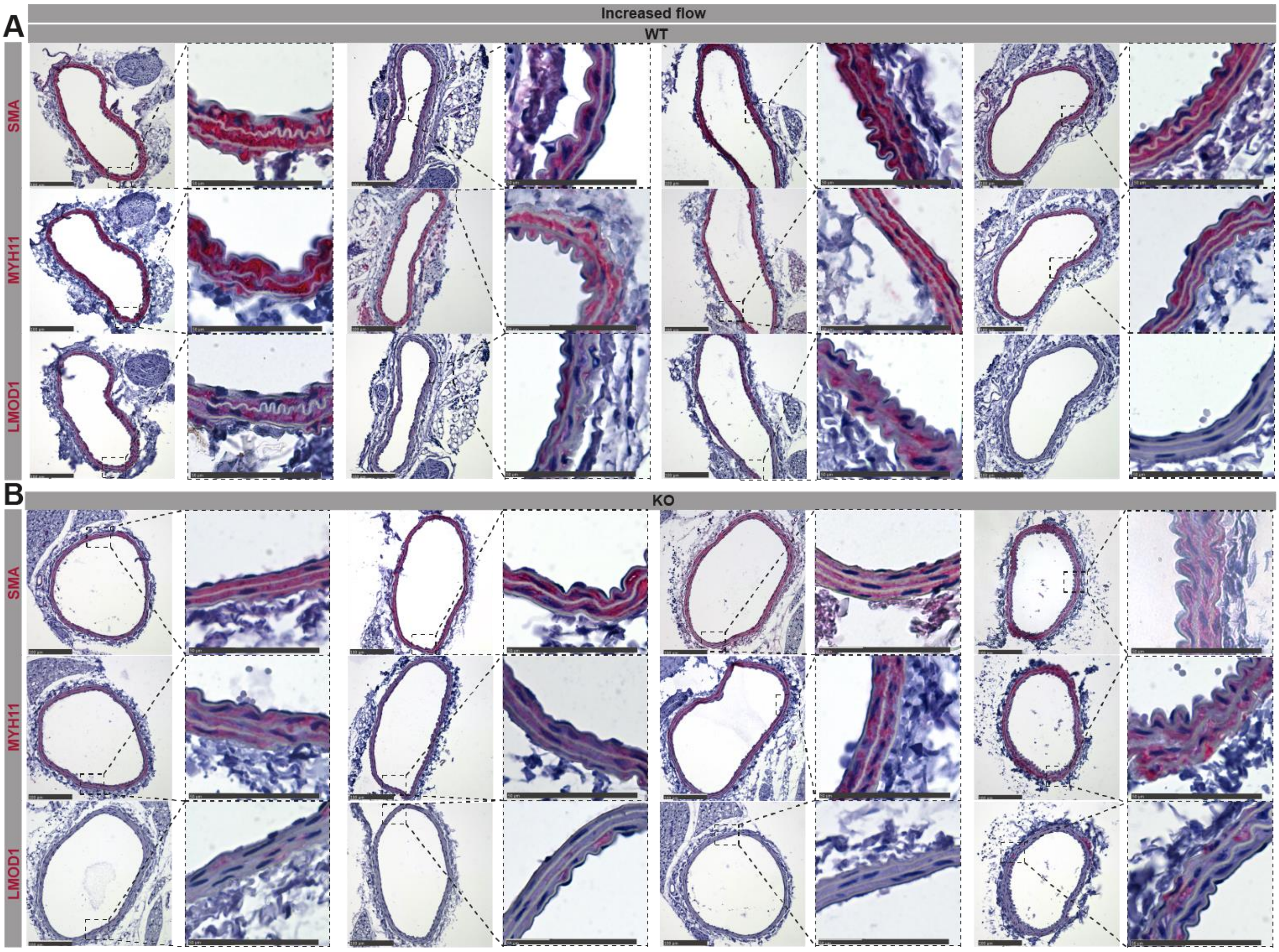

Figure S2. Images from additional mice visualizing the expression of typical smooth muscle cell markers upon increased flow in the right common carotid artery. Images of the right CCA stained for Smooth Muscle alpha-Actin (SMA), Myosin Heavy Chain 11 (MYH11) and Leiomodin-1 (LMOD1) from A) WT and B) KO mice under increased flow conditions (WT n=4, KO n=4). KO= Pcsk6$^{-/-}$ mice. WT= C57Bl/6J mice.
